# Supplementary material for: Psychological price perception may exert a weaker effect on purchasing decisions than previously suggested: Results from a large online experiment fail to reproduce either a left-digit or perceptual-fluency effect
Source: PLoS One. 2022 Aug 18;17(8):e0270850. doi: 10.1371/journal.pone.0270850 (PMC9387778; doi:10.1371/journal.pone.0270850)
Supplement: S1 File — The supporting information contains the results for the 124 participants who purchased one or more of the attention-checks. (DOCX) [file pone.0270850.s001.docx]

# Supporting information: main results for the excluded participants

This appendix contains results for the 124 participants who purchased one or more of the attention-check tickets. These participants had a mean age of 40.2 years; 40.3% was female and 57.3% was male (2.4% selected ‘other’ or chose not to disclose their gender), 62.1% had a Bachelors degree or higher, 82.2% had an annual income over $30,000 (with 41.9% earning more than $60,000). Excluding the attention checks, participants made a total of 2232 purchasing decisions.

Table S1 presents the main results of the two binomial mixed-effects regressions in both of which the dependent variable was a Boolean variable denoting a ticket purchase (1=Yes, 0=No). As in the main text, both models included participant-level random intercepts as a random effect and the treatment variables as fixed effects. In the first model, we tested the main effect of three price-ending treatments by including two dummies coding for the just-below and rounded price treatments. The second model additionally included the price level treatment (a single dummy to denote the low price treatment) and the interaction between the price level and the price-ending treatment. As in the main text, these analyses did not yield a significant difference between the control price-ending treatment and either of the treatment dummies.

**Table S1. Mixed-effects regressions for the excluded participants**

|  | *DV: ticket bought (1=yes)* | |
| --- | --- | --- |
|  | (A1) | (A2) |
| Just-below Dummy | 0.068 | 0.066 |
|  | *(0.123)* | *(0.18)* |
| Rounded Dummy | 0.199 | 0.151 |
|  | *(0.124)* | *(0.181)* |
| Low Price Level Dummy |  | **-0.621***** |
|  |  | *(0.174)* |
| Just-below X Low Price Level |  | 0.006 |
|  |  | *(0.246)* |
| Rounded X Low Price Level |  | -0.097 |
|  |  | *(0.248)* |
| Constant | **0.922***** | ***1.249****** |
|  | *(0.143)* | *(0.171)* |
| Participants | 124 | 124 |
| Observations | 2232 | 2232 |
| Log Likelihood | -1270.1 | -1254.6 |
| AIC | 2548.2 | 2523.2 |
| BIC | 2571.1 | 2563.2 |

*Regression results for the 124 participants who were excluded from the main analysis for failing one or more attention-checks. Both models estimated the best-fitting logistic curve to predict the probability that participants purchased a lottery ticket (1 = yes). Each model included participant-level random effects. In the first model we included dummies for both experimental treatments as fixed effects. The second model additionally included fixed-effects dummies for the low price level and the interaction between the price level dummy and the two experimental conditions. Note: *p<0.1; **p<0.05; ***p<0.01.*

Figure S1 presents a graphical depiction of the mean purchasing rates per price-ending treatment, for the full sample and both price level treatments respectively. Compared to the sample outlined in the main text, the excluded participants had an overall higher propensity to purchase lottery tickets. However, as in the main sample we did not observe a substantial difference between the three price-ending treatments. While there was a small increase in purchasing rates for the two experimental treatments (68% for the just-below treatment and 70.3% for the rounded treatment, versus 66.8% in the control treatment), this difference in purchasing rates was minor, and did not exceed chance probability (model A1; p > 0.05 for both treatment dummies).


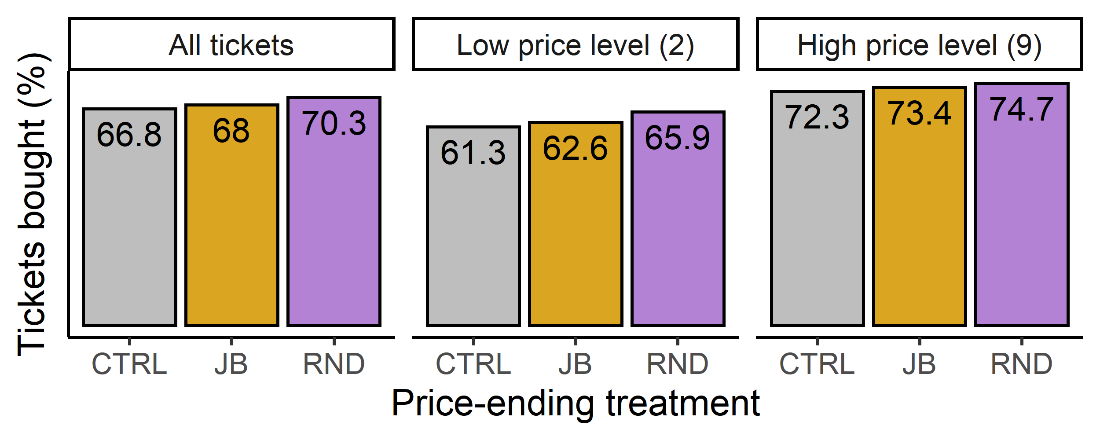


**Fig. S1 -** **results for the excluded participants do not indicate a difference in purchasing rates (y-axis) for the three price-ending treatments*.*** **A:** purchasing rates across both price levels**. B:** purchasing rates for the low price level. **C:** purchasing rates for the high price level. CTRL = control prices, JB = just-below prices, RND = rounded prices.
