# Supplementary material for: Psychological price perception may exert a weaker effect on purchasing decisions than previously suggested: Results from a large online experiment fail to reproduce either a left-digit or perceptual-fluency effect
Source: PLoS One. 2022 Aug 18;17(8):e0270850. doi: 10.1371/journal.pone.0270850 (PMC9387778; doi:10.1371/journal.pone.0270850)
Supplement: Attachment — Submitted filename: Response to reviewers.pdf [file pone.0270850.s002.pdf]

Response to reviewers

The reviewer on the original submission (PONE-D-20-30777) outlined a number of limitations in the original experimental design. These comments could be categorized into three main areas of concern: flaws with the experimental methodology, ambiguities and limitations in the exclusion criteria and limitations in the statistical methodology, including a lack of preregistered hypotheses

When reflecting upon these limitations all authors agreed that both the original design and the data analysis contained a number of irreparable shortcomings. We hence saw it best to re-design the original design entirely, electing to instead build upon a fresh start. As a result, we have decided to scrap the original experiment in its entirety and instead to re-design the original experiment from the ground up. Below is an overview of the changes made to the original methodology, exclusion criteria and data analysis. We feel that these changes have had a strongly positive influence of the overall quality of the manuscript, and we hope that you will share this evaluation.

Changes to the experimental methodology

In the original experiment, participants decided whether or not to purchase each of three lottery tickets. The contents of these tickets differed between the three price ending treatments (prices ending at a rounded X.00 price, a just-below [X-1].99 price or a control price X.01), and between three price-level treatments (X being equal to 1, 5 or 10).

All reviewers noted the overly complicated nature of the experimental design. We believe that this lack of clarity originated from the wieldy and overly complicated between-subjects nature of the original experiment. By re-designing the experiment, we set all of the main experimental treatments to a within-subject design, allowing for a much-reduced complexity in both the empirical design as well as the corresponding statistical analysis. Moreover, this simplified within-subjects design additionally allowed for an increased clarity in the written text of the manuscript.

R1 previously raised a concern with the arbitrary selection of the baseline condition. In the original manuscript we set the baseline (control) price-ending treatment at X.01 (versus X.00 for the rounded condition and [X-1].99 for the just-below condition. R1 argued that the selection of this 01-ending price lacked a clear theoretical argumentation and appeared to be selected on an arbitrary basis. In the new experiment we instead allowed for a wider range of control prices. Instead of a single price ending, we randomized the ending of the control treatment on a between-subjects basis, such that the control tickets ended in a price ranging from X.01 to X.14 (excluding the prices of X.05 or X.09, as these may be argued to be partially induce a perceptual fluency effect or a partial left-digit effect respectively).

In the original manuscript we used three price-level treatments (1, 5 and 10). R2 argued that the selection of the three price levels (1, 5 and 10) was unfortunate, as the number of leading digits changed between the treatment's differences, thereby inducing more than a pure level effect. More-over, in a purely technical perspective, the first leading digits for the high price level (10) was higher in the just-below treatment (9.99, corresponding to a leading digit of 9) than for the other two treatments (10.00 and 10.01, corresponding to leading digits of 1). We addressed these changes in the new experiment by including two different price-level treatments: low (2) and high (9). These new price levels did not include a change in the number of leading digits. Additionally, the high price level treatment no longer had a higher value first leading digit for the just-below treatment (now 8.99, corresponding to a leading digit of 8) than for the other two treatments (now 9.00 and 9.01, both corresponding to a leading digit of 9).

Changes to the exclusion criteria

In the original experiment we excluded a large number of participants based on inconsistent choice patterns (that is, participants who decided to purchase objectively less attractive lotteries, whilst deciding not to purchase more attractive lotteries). R1 argued that this exclusion rule was not pre-registered and may have been selected on a post-hoc basis. In the new experiment we adopted a pre-registered and systematized approach to ensure a sufficient quality of data through the inclusion of three attention-check tickets. These tickets were construed such that their high outcome had a payoff equal to 0.9 times the ticket price. As these tickets returned a negative payoff even in the best-case scenario, we assumed that no attentive participant would be interested in purchasing these tickets. Consequentially we excluded any participants who purchased one or more of these tickets from further analysis. Notably, all reported results are robust to the inclusion of these participants.

Changes to the statistical methodology

A main concern raised with the analyses of the original manuscript revolved around the exploratory nature of the analyses. All authors agreed with these concerns. In the new experiment we pre-registered any expected effects based on existing theoretical arguments. Additionally, we abstained from reporting p-values for all statistical tests which were not pre-registered.

R1 raised a concern with the nature of the statistical strategy employed in the original manuscript. Here we previously collapsed each participant's purchasing decisions into a single value: the number of tickets purchased. This analysis strategy ignores potential differences between different tickets and does not directly estimate the per-lottery purchase rate. R1 therefore suggested an alternative statistical methodology through the use of logistic regressions. The new experimental methodology was explicitly designed with such a logistic regression in mind. Eliciting all experimental treatments on a within-subjects basis allowed us to estimate two mixed-effects logistic regression models.

R2 further raised a concern with the pooling nature of our statistical tests. That is, in the original manuscript we tested the left digit effect by comparing the mean number of tickets purchased by tickets in the just-below treatment versus the mean number of tickets purchased in the other two price-ending treatments. Conversely, we tested the perceptual fluency effect by comparing the mean number of tickets purchased in the rounded price-ending treatment versus the mean number of tickets purchased in the other two price-ending treatments. R1 argued that this pooling of the data may have induced a distortionary effect on the statistical tests. The authors agreed with this assessment. In the new experiment, we tested both the LDE and the PFE simultaneously by including two treatment dummy variables in our mixed-effects logistic regression models.
